# Surgical Approach and Considerations for Compressive Thoracic Intraspinal Osteochondroma in Familial Hereditary Multiple Exostosis

**DOI:** 10.3390/diseases12070165

**Published:** 2024-07-19

**Authors:** Corneliu Toader, Antonio-Daniel Corlatescu, Nicolaie Dobrin, Razvan-Adrian Covache-Busuioc, Horia Petre Costin, Alexandru Vlad Ciurea

**Affiliations:** 1 Department of Neurosurgery, Carol Davila University of Medicine and Pharmacy, 8 Eroii Sanitari Blvd, 050474 Bucharest, Romania; corneliu.toader@umfcd.ro (C.T.); antonio.corlatescu0920@stud.umfcd.ro (A.-D.C.); razvan-adrian.covache-busuioc0720@stud.umfcd.ro (R.-A.C.-B.); horia-petre.costin0720@stud.umfcd.ro (H.P.C.); prof.avciurea@gmail.com (A.V.C.); 2 National Institute of Neurology and Neurovascular Diseases, 10-12 Berceni St., 077160 Bucharest, Romania; 3 “Nicolae Oblu” Clinical Hospital, 700309 Iasi, Romania; 4 Sanador Clinical Hospital, 9 Sevastopol St., 010991 Bucharest, Romania; 5 Department of Medical Sciences, Honorary Member of The Romanian Academy, 010071 Bucharest, Romania

**Keywords:** osteochondroma, hereditary multiple exostosis, spinal surgery, EXT1, EXT2

## Abstract

Introduction: Hereditary multiple exostosis or hereditary multiple osteochondromas is a very rare clinical condition. Usually, these lesions tend to occur in the pediatric population, remaining silent until adulthood. Moreover, current studies show a small prevalence in the male population. The osteochondromas usually occur at sites with great bone activity and turnover, such as the diaphysis or metaphyseal plates (especially in children) of long bones. Their appearance in short bones (such as vertebrae) is very rare. Case presentation: We present a case of familial HME in a 53-year-old female patient with a very uncommon clinical description of the disease. The patient presented at our hospital with Frankel D-type paraparesis, with multiple osteochondromas (located at the right humerus, bilateral femurs, right tibia, and hip joints, besides the numerous ones over the spinal column) and urinary incontinence. She was suffering from bilateral coxarthrosis and gonarthrosis, which limited severely the range of her movements. An early menopause status was brought into consideration by the patient, being installed circa 15 years before, at 38 years old. She was currently in treatment with bisphosphonates for her concomitant osteoporosis. Conclusions: Despite the relatively rare nature of the disease, it may be an important concern for the patient’s quality of life. Intraspinal processes may trigger paraparesis or other neurological statuses, which may require a surgical treatment. The nature of the lesions is usually benign and do not require further radio- or chemotherapy.

## 1. Introduction

Hereditary multiple exostosis (HME), a genetic disorder with an autosomal dominant transmission, exhibits an incidence of 1 in 50,000 people, or 0.9–2 in 100,000. This condition is more common in the Caucasian population, with a notably higher prevalence in individuals from Canada and Guam Island. HME demonstrates a high penetrance rate of 96–100%, indicating that nearly all individuals with the associated gene mutation display varying degrees of symptoms, highlighting its familial nature. Studies indicate a higher penetrance in males, though they often exhibit milder symptoms compared to females [1]. While earlier research suggested an equal distribution across genders, more recent findings indicate a slight male predominance, with a male-to-female (M/F) ratio of 1.5:1 [2]. Approximately 96% of HME patients are within the pediatric age group, ranging from 2 to 12 years old [3]. Research has identified that HME results from the loss of function in two separate genes: exostosin-1 (EXT1), located on chromosome 8, and exostosin-2 (EXT2), located on chromosome 11 [4,5]. However, the specific pathological process leading to the development of multiple osteochondromas is not completely understood. The number and distribution of lesions vary widely, potentially affecting any part of the skeleton.

Although the lesions in hereditary multiple exostosis (HME) are benign, they can significantly impair the patient’s quality of life. These include aesthetic deformities such as genu valgum, digital and humeral asymmetries, coxa valga, coxa magna, and scoliosis, as well as neurological deficits like radial or ulnar nerve palsy, tetraparesis, or paraparesis. Complications can extend to bursitis, particularly if a synovial joint is involved, fractures, and neurological deficits, especially in cases of intraspinal overgrowth. While many lesions remain clinically asymptomatic, some can lead to severe morbidity, intense pain, restricted joint movements, and impingement of neurovascular structures. In HME, multiple cartilage-capped exostoses typically emerge during childhood and ossify upon the completion of skeletal growth [6]. These bony protrusions are benign but have a potential for malignant transformation into chondrosarcomas (CHSs) in approximately 3.9% of patients. CHS transformation generally occurs before the age of 40, with 87% of cases affecting the appendicular skeleton. The most common sites for CHS include the pelvis, scapula, proximal femur or spine, and ribs. Although CHS is usually low-grade, its proximity to major neurovascular bundles often complicates radical surgical excision. Research has indicated that individuals with EXT1 mutations have a 1.5–2 times higher risk of malignant transformation compared to those with pathogenic variants in EXT2 [7,8].

Arthritis is a frequent manifestation of this disease. In a small percentage of cases, approximately 1–5% in adults, HME can progress to malignancy, developing into osteosarcomas or chondrosarcomas, which are associated with poor outcomes and reduced quality of life [2,9]. The most severe complication is the potential for malignant transformation, affecting up to 5% of adult patients [10]. Osteochondromas may be deeply situated, making surgical intervention difficult or impossible. In many instances, the lesions are left untreated, but when they severely affect the quality of life, surgical removal becomes essential.

## 2. Case Presentation

We present a case of hereditary multiple exostosis in a 53-year-old female patient who had an osteochondroma located within the spinal canal at the thoracic level of the vertebral column. She was diagnosed years before her surgical operation and had a positive family history, with six siblings also affected by the disease, exhibiting multiple exostoses in various parts of their bodies. The patient herself had at least two other exostoses located at different levels of her vertebral column. She came to our hospital with Frankel D-type paraparesis, making walking nearly impossible without support, and experienced sensory loss localized at the T10 dermatome. Notably, the patient had experienced early menopause, which is relevant due to its potential connection with genetic abnormalities, bone health, and hormonal influences leading to low estrogen levels and reduced bone density.

**Figure 1:**
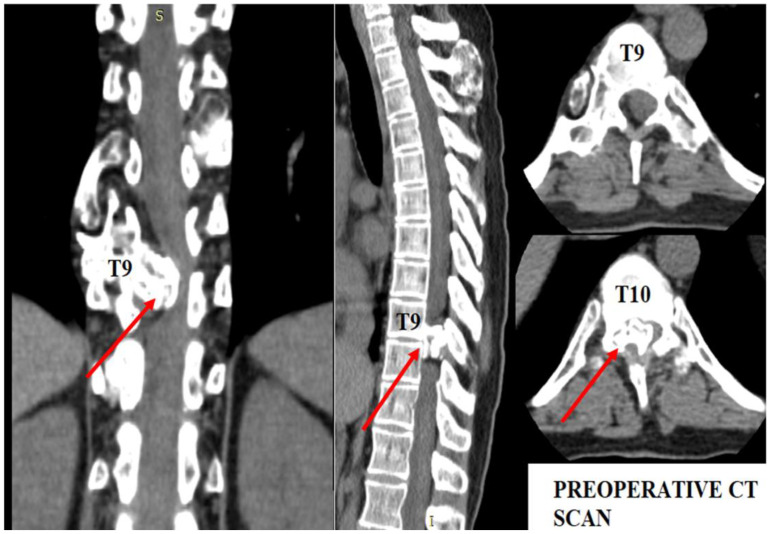
Preoperative CT scan. Note the intracanalar localization determined by the osteochondroma.

The patient underwent diagnostic evaluations with both MRI and CT scans (Figure 1), with the CT scan being particularly crucial for assessing bone and cartilage tissues, which are primarily affected in this disease. The imaging revealed an intracanalicular lesion at the T9–T10 level, compressing nerve roots and constricting the spinal cord. Additionally, she had osteochondromas at the right humerus, bilateral femurs, right tibia, and hip joints, along with numerous others along the spinal column. She also suffered from bilateral coxarthrosis and gonarthrosis, which severely limited her range of movement.

This case highlights the complexities and challenges of managing hereditary multiple exostosis, especially when complicated by neurological symptoms and significant comorbidities such as early menopause and severe joint issues.

As shown in Figure 1, the osteochondroma occupied roughly three-quarters of the whole caliber of the medullary canal. Using the CT scan, the mass showed a bony signal with a homogenous composition. The shift of the spinal cord was very relevant, as the patient could not walk, or maintain orthostatism or sphincter control.

The patient was scheduled for surgical intervention a few days after the CT scan. The surgical team opted for a bilateral laminectomy at the T9–T10 level to reduce the stenosis and remove the osteochondroma which grew both outside and inside the spinal canal, occupying circa 80% of the whole caliber of the vertebral canal.

**Figure 2:**
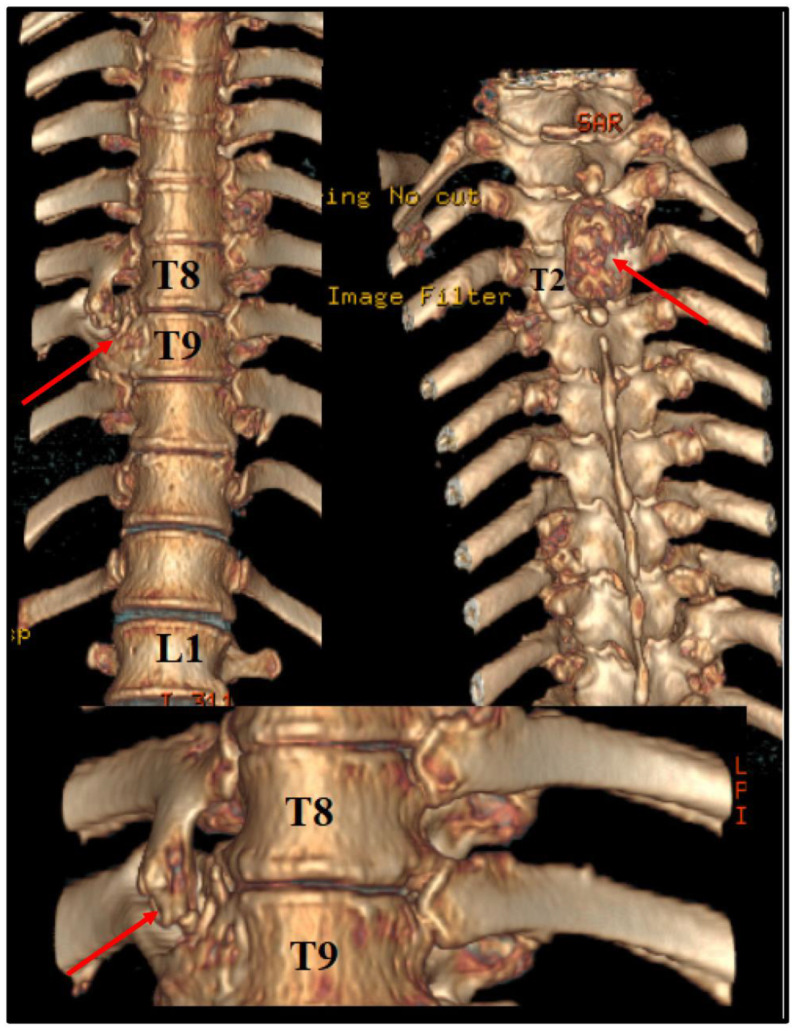
Preoperative 3D CT scan. Note the outer margins of the osteochondroma, along with another one, localized at T2 level, which was neurologically silent.

Before the intervention, we asked for a 3D CT reconstruction, which helped to appreciate the exterior aspect of the vertebral column, as well as to acknowledge the outer extensions of the exostosis (Figure 2).

We used a standard thoracic approach for the surgery. The patient was positioned prone, parallel to the ground. A 10 cm paravertebral skin incision was made over the spinous processes of the T9–T10 vertebrae. Laminectomy, primarily on the right side, was performed to access the spinal canal, and the surgical microscope was brought into the field. The formation was hard to the touch, resembling bone tissue, and involved the dentate ligament, posterior longitudinal ligament, and yellow ligament. The thoracic spinal cord was compressed and shifted contralaterally, with its width reduced by up to 80% at the site. The tumor was removed using Kerrison forceps (sizes 3, 4, and 5), with special care taken to avoid CSF fistulas. Under the optic microscope, a high-speed surgical drill was used to remove the remaining osteochondroma. After gross removal, meticulous hemostasis was performed. Once fully decompressed, the spinal cord returned to its normal size. Postoperative recovery was excellent; the patient was admitted to the ICU for two days but did not require any special interventions or treatments.

Seven days after the surgery, the patient was discharged. No signs of CSF fistulas were detected. At discharge, the patient could walk normally, her paraparesis had significantly improved, and her sensory deficits had resolved. Orthostatic function was within normal ranges, and there were no signs of urinary incontinence.

## 3. Results

**Figure 3:**
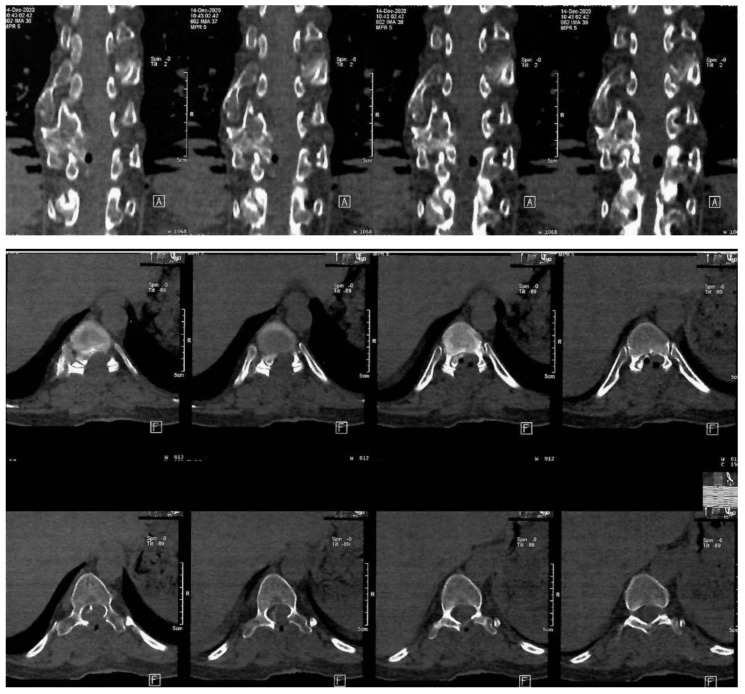
Postoperative thoraco-lumbar native CT scan. A: indicate the anterior (front) side of the body relative to the position of the cross-sectional slices being viewed, F: indicates the orientation of the patient’s feet during the scan.

The mass was sent to histopathology in our clinic. According to the report, fragments of osteochondroma were discovered, which was compatible with HME. The operation was a great success, with a considerable improvement of the neurological status. The Frankel D paraparesis remitted in about a week. A CT scan was performed 6 days after surgery. The Frankel D type is characterized by the preservation of some degree of motor function below the level of the spinal cord injury, allowing the patient to have active movement, although it may not be sufficient to support all activities of daily living independently. Sensory function is also preserved to some extent below the level of the injury, enabling the patient to feel touch and other sensations, although the sensitivity may be reduced. Patients with Frankel D can usually walk, but they may require assistance or aids such as braces, crutches, or walkers, as their muscle strength is often not sufficient to support full, unassisted mobility. Note the important enhancement of the spinal column bio-architecture, plus the increase in the vertebral canal width (Figure 3).

The postoperative thoracal CT scan showed visible laminectomy, with marked enlargement of the vertebral canal caliber. The postoperative CT scan confirmed the total resection of the osteochondroma.

**Figure 4:**
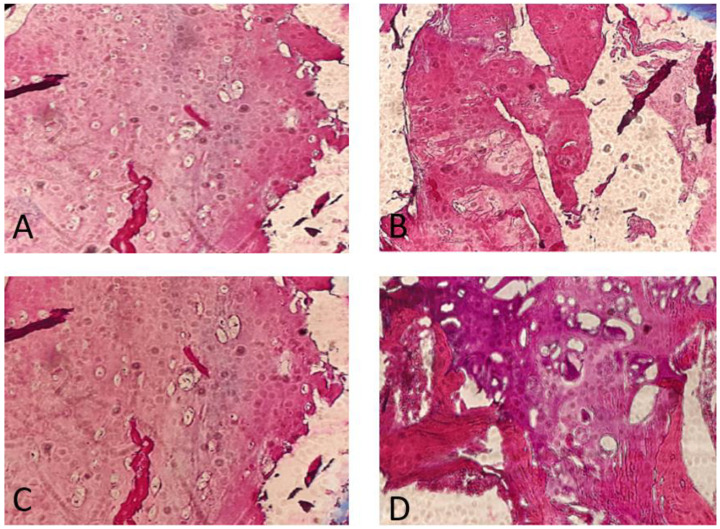
Anatomopathological findings. Note the great proliferation and activity of the chondrocytes. Osteochondroma, H&E stain. (**A**) Cross-section through the lesion showing a cap of osteochondroma with the orderly arrangement of chondrocytes in a linear pattern, similar to that seen in the process of normal endochondral ossification (4×). (**B**,**C**) Detailed images of focally calcified hyaline cartilage (10×). (**D**) Cancellous bone with marrow elements (10×).

The histopathology findings were studied in our hospital. They showed a high proliferative cartilage tissue, with bone fragments (Figure 4).

The patient was followed-up at three months. The first medical examination after surgery brought a very satisfied patient. She could walk, with sporadic use of a helping stick, and had good sphincter control.

## 4. Discussion

This disease has an autosomal dominant pattern, being a predominantly inherited disease. It alters the physiological growth of the normal cartilage–bone interface, and it develops sites of exostosis in the growing cartilage plates, hence it is more common in long bones (e.g., the femur and humerus). These formations include a normal bony pedicle, covering the proliferative cartilaginous tissue, which will finally cause the development of the exostoses [11,12]. Other uncommon sites were described, as well as the anterior aspect of the lumbar vertebral column, with abdominal extension of the lesion. Intracanalicular exostosis remains a rarity in the literature. Most cases are present among the pediatric population, with up to 95% of cases having its incidence before 12 years of age. Nonetheless, cases like our own, affecting the adult population, may need a surgical approach, especially due to the symptoms conferred by the location of the lesion. If the exostoses appear at the surface of the bone (e.g., the femur, humerus, or ribs), the deformation may cause social stigma and ostracization, which may further induce the patient to seek medical advice. In our case, the lesion determined a neurological status, which was progressive and invalidating, and sphincter dysfunction.

Recent literature emphasizes the critical role of heparan sulfate (HS) biosynthesis in the pathogenesis of hereditary multiple exostoses (HMEs). The EXT1 and EXT2 genes encode glycosyltransferases essential for HS chain polymerization. Defects in these genes disrupt HS biosynthesis, which, in turn, affects several key signaling pathways, including fibroblast growth factor (FGF), bone morphogenetic protein (BMP), hedgehog, and Wnt signaling pathways. These pathways are crucial for normal chondrocyte differentiation and proliferation, explaining the development of exostoses in HME patients [13,14,15].

The disease tends to develop progressively during childhood and adolescence, ceasing growth with the maturation of the epiphyseal plates [16]. The average count of exostoses per patient is between 15 and 18, but this number can vary significantly, ranging from 2 to as many as 172 osteochondromas [17]. HMEs are more likely to arise in bones that form through endochondral ossification, particularly in long bones. The clinical severity of the disease is influenced both by the total number of exostoses and their size and shape. Osteochondromas usually grow outward from the epiphysis and can be either pedunculated or sessile in nature. The term ‘pedunculated’ describes a slender outgrowth with a narrow stalk, which is more prone to causing local trauma due to irritation of the surrounding tissues.

The variability in the clinical presentation of HME, such as the number and size of osteochondromas, can be attributed to the different mutations within the EXT1 and EXT2 genes. Patients with EXT1 mutations often exhibit more severe phenotypes, including a higher number of osteochondromas, greater limb deformity, and a higher risk of malignant transformation compared to those with EXT2 mutations. This genotype–phenotype correlation is critical for understanding disease prognosis and tailoring patient management. The products of the EXT1 and EXT2 genes form a complex in the Golgi apparatus, which is crucial for the production and function of the glycosyltransferase enzyme. This enzyme is responsible for the polymerization of heparan sulfate (HS). Therefore, mutations in either EXT1 or EXT2 can cause the disease, because the Golgi complex cannot function properly without these components [18,19].

Alongside imaging techniques, genetic testing offers the highest specificity for diagnosing hereditary multiple exostoses (HMEs). This testing can detect the disease even before symptoms appear, enabling the planning of long-term monitoring [20]. Genetic counseling is strongly advised for adults with HME who are considering parenthood, as well as for parents of children diagnosed with HME, to assess the risk of transmitting the disease to future offspring. Molecular testing is also crucial in the postnatal screening of children born to affected individuals and their at-risk relatives. Currently, only two genes, namely, EXT1 and EXT2, are associated with HME, and routine screening focuses on identifying point mutations, intragenic deletions, duplications, or occasionally more complex rearrangements in these genes. Techniques used for screening causative mutations include PCR followed by Sanger sequencing, MLPA (using MRC Holland kits), quantitative PCR, and, where available, next-generation sequencing-based methods [21].

Intracanalicular exostosis, although rare, poses significant clinical challenges due to its potential to cause neurological symptoms, as demonstrated in the current case. Literature reports suggest that while most exostoses are asymptomatic, those located in anatomically critical regions, such as the spine, can lead to severe complications, including spinal cord compression and neurological deficits. This underscores the necessity for regular monitoring and timely surgical intervention in symptomatic cases.

Hereditary multiple exostosis is an inherited disorder characterized by multiple osteochondromas. Clinical manifestations are usually absent before 2 years old but discovered before age 10. The main complaint is the discovery of single or multiple hard painless masses near joints. The distribution is usually bilateral and may be symmetrical, leading to dwarfing and deformities. Malignant degeneration into chondrosarcoma occurs in up to 5% of the whole cases. It is therefore important to monitor all cases of HME, especially if the patient complains of pain or the growth of an osteochondroma [7].

The patient’s familial history was also relevant, since 6 out of 7 of her brothers had manifestation of HME, showing off the high penetration rate of such a condition. However, the lesions were clinically silent. In the literature, a greater rate of malignant transformation was more representative in the pediatric population, with up to 25% of cases degenerating into malignant tumors. This detail is very important, because the greatly proliferative tissue may invade the surrounding tissues and create distant metastases.

Malignant degeneration in HME patients is a rare occurrence. It usually presents as a differentiated chondrosarcoma originating from the cartilage cap, though, in rare cases, dedifferentiated chondrosarcomas or osteosarcomas can develop from the bone base. Clinical suspicion arises when the cartilage cap exceeds 1.5–2 cm in thickness or when there is significant growth of the exostosis in adults. Approximately 2 to 4% of HME patients experience malignant degeneration, with a yearly risk of 0.1% for those aged 30–50. Other studies indicate that 0.9% of patients diagnosed with chondrosarcoma have a lifetime risk of developing malignant degeneration, ranging from 1 to 6% or 1 to 5% [22,23].

In the case of hereditary multiple exostoses (HMEs), the disorder impacts the cellular biosynthesis of heparan sulfate, a key component for the functioning of various metabolic pathways. This includes pathways such as FGF, BMP, hedgehog, and retinoid signaling. Any of these pathways could potentially be targeted for treatment strategies [6,10]. Even though the lesions are usually benign in most cases, some malignant potential is observed, especially in the pediatric patients (up to 25% of cases in the most recent studies).

## 5. Conclusions

The great variability of the symptoms and signs makes such a disease a great challenge for different surgeons, not only neurosurgeons. Orthopedic cases have been described in the medical literature, having abdominal, thoracic, and cervical extensions. Most operated cases had aesthetic issues. Our case presented a neurological status due to the intracanalicular occupation of the osteochondroma, with a subsequent shift of the spinal cord. Signs of urinary incontinence, as well as paraparesis or other neurological signs, made the surgical ablation necessary. Histopathological assays are also mandatory to confirm the disease. Moreover, a multidisciplinary approach must be considered for lesions that protrude in many different compartments. It is important to remind that these lesions are due to a great chondrocyte activity rather than osteoblasts (hence the potential malignant transformation). We emphasize the difficulty in diagnosis, as well as its differential diagnosis, and to increase attention over a medical issue which may be trivial to the patient itself. The bony structure may be difficult to resect totally due to its strong texture.

In conclusion, hereditary multiple exostosis (HME) is an inherited disorder with an autosomal dominant pattern, primarily affecting the normal growth of the cartilage–bone interface. This condition leads to the development of exostoses, particularly in long bones like the femur and humerus, but can also occur in rare sites such as the lumbar vertebrae. While the majority of cases present in the pediatric population, HME can also affect adults, sometimes necessitating surgical intervention due to symptomatic lesions. The products of the EXT1 and EXT2 genes are crucial for the production of heparan sulfate (HS), and mutations in these genes disrupt this process, affecting key signaling pathways essential for chondrocyte differentiation and proliferation.

Regular monitoring is crucial, especially if the patient experiences pain or the growth of an osteochondroma. Overall, while HME is primarily a benign condition, its potential for malignant transformation, particularly in pediatric patients, underscores the importance of vigilant monitoring and timely intervention. This comprehensive understanding of HME, including its genetic basis, clinical presentation, and management strategies, is vital for improving patient outcomes and quality of life.

## Data Availability

No new data were created or analyzed in this study. Data sharing is not applicable to this article.

## Abbreviations

CT	computed tomography
HME	hereditary multiple exostosis
HMO	hereditary multiple osteochondromatosis
ICU	intensive care unit
H&E	hematoxylin and eosin coloration
